# ALDOB plays a tumor-suppressive role by inhibiting AKT activation in gastric cancer

**DOI:** 10.7150/jca.83456

**Published:** 2023-07-16

**Authors:** Chaozhong Peng, Xuan Yang, Xiao Li, Zhixue Ye, Jiangming Wang, Wenqing Wu

**Affiliations:** 1 Department of General Surgery, Suzhou Wuzhong People's Hospital, Suzhou 215128, Jiangsu, China.; 2 Department of Gastroenterology, Suzhou Wuzhong People's Hospital, Suzhou 215128, Jiangsu, China.

**Keywords:** Gastric cancer, ALDOB, proliferation, AKT, migration.

## Abstract

**Abstract:** Enhanced aerobic glycolysis has been one of the cancer hallmarks. Cancerous cells develop certain alterations during glucose metabolism for supporting their infinite growth requirement and metastasis. Therefore, targeting metabolism, for instance, crucial glycolytic enzymes, will provide a novel therapeutic strategy to treat cancer. Aldolase B (ALDOB), as a glycolytic enzyme, plays a contentious role in cancers and can either act against the tumor or as an oncogenic enzyme. The precise role of ALDOB in gastric cancer (GC) and the endogenous process is elusive and needs further exploration. This investigation revealed that ALDOB expression was markedly decreased in GC tissues. Furthermore, ALDOB inhibition was notably linked with tumor size, depth of tumor invasion, lymph node metastasis (LNM), tumor node metastases (TNM) staging, and substandard prognosis of GC. The assessment of loss- and gain-of-function indicated that ALDOB inhibited the growth and the migrative ability of GC cells, suggesting its anti-tumor role. Mechanistic studies revealed that ALDOB modulates the AKT signaling pathway. The increase in growth and cells' ability to migrate stimulated by ALDOB inhibition was partially impaired in cells under the influence of AKT inhibitors. The overall data highlights a novel target, the ALDOB/AKT signaling axis for the treatment of GC.

## Introduction

Gastric cancer (GC) is malignant and linked with increased morbidity and death rates, globally 1. About 110,000 new cases and 770,000 deaths have been reported for GC, annually, which accounts for 5.6% of all cancers and 7.7% of total deaths related to cancers 2. Recently some advancements have been made in clinical therapy, however, prolonged survival of patients with terminal cancer is still not achieved. GC has only a 12% of 5-years survival rate 3. The main reason behind this low survival rate is GC recurrence. Therefore, exploring the underlying molecular process responsible for GC development and its progress and new therapeutic targets is urgently needed.

Alterations in energy metabolism are essential for cancer progression 4. Therefore, cancerous cells adapt characteristic alterations in glucose metabolism for supporting infinite growth and metastasis 5. Members of the Aldolase family (ALDOA, ALDOB, and ALDOC) are 4^th^ enzymes in glycolysis. 6. They catalyze the reversible reaction where fructose-1,6-bisphosphate is converted into dihydroxyacetone phosphate and glyceraldehyde-3-phosphate 6, 7. Aldolase B (ALDOB) or liver-type aldolase, is located in the liver and kidneys 8. Abnormalities in ALDOB are associated with various diseases, for instance, hereditary fructose intolerance (HFI), liver cirrhosis, hepatitis, and even cancer 9-11.

Literature has revealed that abnormal ALDOB expression is closely linked with colorectal cancer (CRC) 12-16 and hepatocellular carcinoma (HCC) 17-19. The overexpression of ALDOB in CRC is linked with tumor progression via transition of epithelial-mesenchyme and substandard prognosis 12. ALDOB also enhances fructose metabolism and promotes liver metastases of CRC, suggesting that ALDOB inhibition may reduce liver metastasis growth 15. In contrast, ALDOB inhibits cell migration and acts as a prognostic biomarker for HCC 17. One investigation discovered a novel role of ALDOB, it was indicated that it negatively regulates AKT activation, suggesting its potency as a therapeutic target for treating HCC 19. Thus, ALDOB has controversial roles which depend on the type of cancer. Recently, an investigation showed that ALDOB is markedly reduced in GC tissues and is associated with substandard prognosis 20. However, the precise function of ALDOB in GC cells and its endogenous mechanism remains unknown and need further elucidation.

In the present investigation, it was discovered that ALDOB expression in GC tissues was reduced and it exerted an oncogenic role. Mechanistic analyses revealed that ALDOB stimulated GC cell growth and metastasis by the AKT signaling pathway, which may provide an innovative strategy in GC treatment.

## Materials and methods

### GC tissues and cell lines

The GC tissue specimens were acquired from enrolled patients in the Suzhou Wuzhong People's Hospital for surgery between the period of January 2012 to January 2021. All the participants were informed about the study and then their consent was taken. The study was performed under the Declaration of Helsinki. The GC patients' clinicopathological characteristics are depicted in Table 1. The inclusion criteria were as follows: (1) histological confirmed GC; (2) complete clinicopathological data (including age, gender, tumor size, tumor location, tumor invasion depth, lymph node metastasis (LNM), tumor node metastases (TNM) staging); (3) without coexistence of other tumors and hematological diseases; (4) received radical gastrectomy. The exclusion criteria were as follows: (1) received neoadjuvant chemotherapy or radiotherapy before surgery; (2) histological diagnosed second primary tumor; (3) acceptance of palliative surgery.

GC cell lineages (MKN45 and AGS) were acquired from the Type Culture Collection of the Chinese Academy of Sciences, Shanghai, China, and propagated in RPMI 1640 (Gibco) media augmented with fetal bovine serum (FBS, 10%, Gibco) and streptomycin/penicillin (1%, Invitrogen), in an environment of 5% CO_2_ and 37 °C temperature.

### Kaplan-Meier Plotter

Clinical data were obtained from the Kaplan-Meier Plotter platform (https://kmplot.com), a powerful tool capable to analyze the association between cancer patients' prognosis and gene expression.

### Immunohistochemistry (IHC)

All the tissues initially underwent the protocol of fixation by formalin and then submerged in paraffin before being sliced into 4μm thick fractions. Then these fractions were kept in anti-ALDOB primary antibody (diluted at 1:100; 18065-1-AP; Proteintech) at 4°C overnight, thenceforth 1 h secondary antibody treatment at room temperature. Counterstaining was carried out with Hematoxylin. IHC scores were analyzed independently by two pathologists without prior information about the patient's manifestations. Based on the frequency and intensity of staining, the positive rate was scored as 0 (no staining), 1 (1-25%), 2 (26-50%), 3 (51-75%) or 4 (76-100%), and the staining intensity was graded as 0 (negative), 1 (weak), 2 (intermediate) or 3 (strong). The final score was calculated by the multiple of the intensity and extent score.

### Western blotting assay

Whole cellular proteins were isolated by SDS-PAGE after extraction and then transmitted from the gel to PVDF membranes. In 5% milk, the membranes were blocked and then left overnight in the primary antibody at 4°C before secondary antibody treatment. Bands were visualized by chemiluminescence (Tanon, China). Primary antibodies utilized in this investigation were: anti-ALDOB antibody (at 1:1000 dilution; 18065-1-AP; Proteintech), anti-p-AKT (Ser473) (at 1:1000 dilution; #4058, CST), anti-AKT (at 1:1000 dilution; #9272, CST) and anti-β-actin (at 1:1000 dilution; 66009-1-Ig; Proteintech).

### Construction of stable cell lines

GC cells with stable expression of short hairpin RNA (shRNA) specific to ALDOB and human ALDOB encoding plasmids were produced to decrease and increase ALDOB, respectively via a lentivirus protocol (GeneChem, Shanghai, China). The ALDOB shRNA target sequence is 5'-ATT ACC TCT GGT TCA ACAAT-3'.

### Colony formation assay

Cells were propagated in a 6-well plate at 1,000 /well concentration and 37°C for 7-10 days. After incubation cell fixation was performed using 4% paraformaldehyde (Beyotime, Beijing, China), followed by 0.1% crystal violet (Beyotime, Beijing, China) staining. Colonies comprising >50 cells/well were quantified.

### Transwell migration assay

Re-dissolved cells in FBS-negative RPMI 1640 media were propagated in the upper chamber the of transwell (Corning, USA). In the lower chamber, RPMI 1640 media augmented with 10% FBS was added. Lower surface transferred cell fixation was carried out by paraformaldehyde (4%), followed by crystal violet staining (0.1%).

### Statistical analysis

All the results were depicted as means ± standard errors (SEM). A two-tailed Student's *t*-test was applied for analyzing the statistical variations between the two data sets. IHC results were evaluated via a chi-square test, propensity score matching (PSM) was performed to reduce selection bias. *P* < 0.05 was deemed a value with statistical importance.

## Results

### ALDOB is decreased in GC tissues and low ALDOB expression is correlated with poor survival

**Figure 1:**
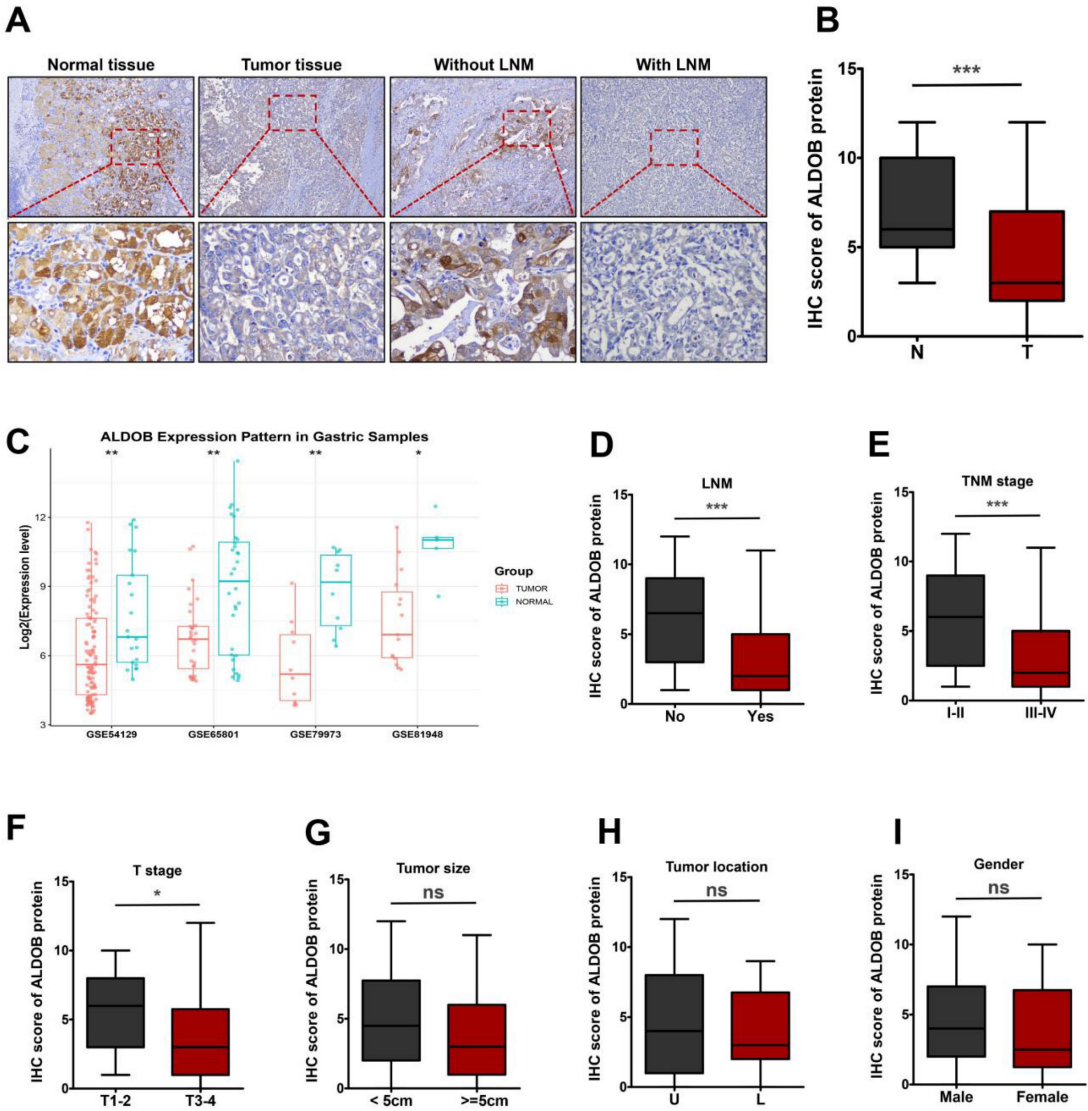
ALDOB is down-regulated in GC tissues. A. IHC assessment of ALDOB in GC and NT. B. IHC scores for GC tissues' ALDOB levels (T) and NT (N). C. Gene chip data were obtained and compared from 4 GEO datasets. D. IHC scores for GC tissues' ALDOB levels in the presence and absence of LNM. E-I. IHC scores for GC tissues' ALDOB levels at different TNM stages (E), different T stages (F), different sizes (G), different locations (H), and different genders (I). ns, non-significant, *P < 0.05 , **P < 0.01, ***P < 0.001.

**Figure 2:**
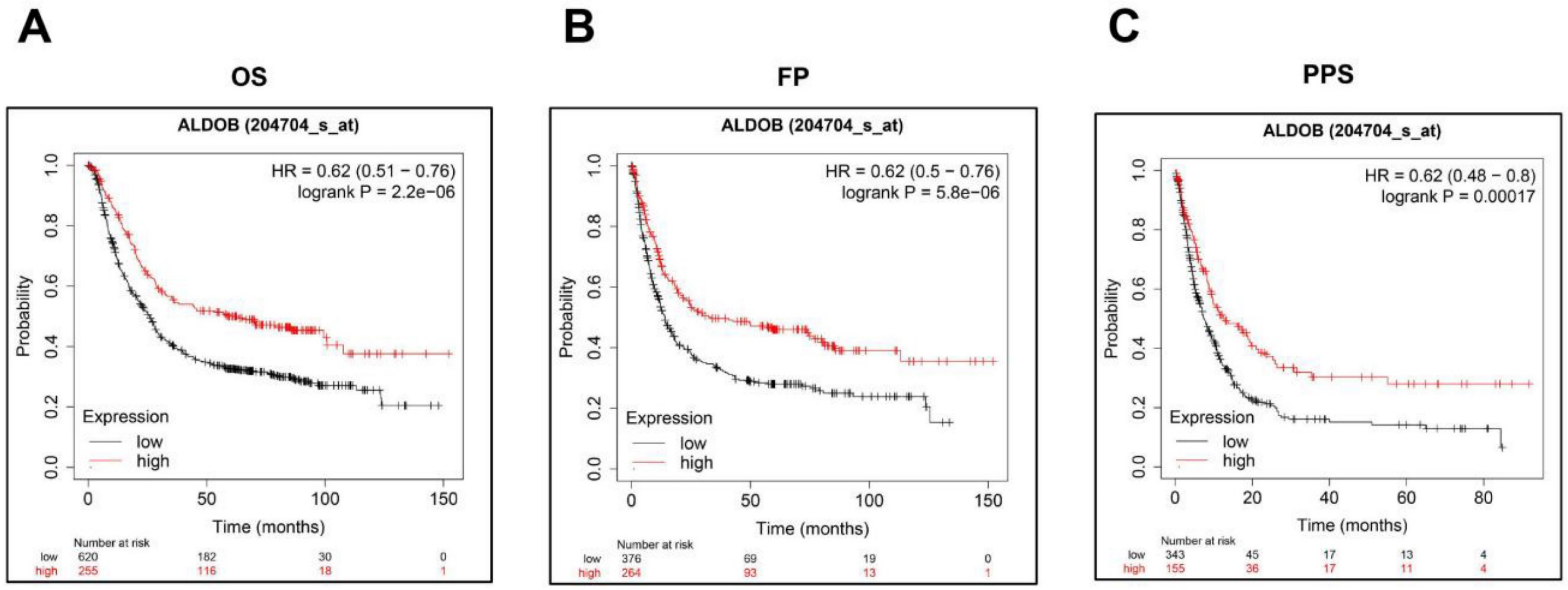
Low ALDOB mRNA expression in GC is associated with reduced survival. (A-C) Kaplan-Meier curve for the OS (A), FP (B), and PPS (C) of GC patient's data acquired from Kaplan-Meier plotter databases.

To determine the function of ALDOB in GC, its levels in tumor and normal tissues (NT) were evaluated. IHC analysis revealed that ALDOB levels were markedly reduced in GC tissues than in the NT (*P* < 0.001, Figure 1A and 1B). To validate the expression of ALDOB in gastric tumorigenesis, we analyzed ALDOB mRNA expression with the public GEO (Gene Expression Omnibus) databases (GSE54129, GSE65801, GSE79973 and GSE8194), results revealed that ALDOB mRNA level was down-regulated in gastric cancer tumors compared with their normal counterparts, which is consistent with our IHC analysis. Subsequent analysis revealed that ALDOB levels in GC tissues with lymph node metastasis (LNM) were decreased than in non-LNM GC tissues (*P* < 0.001, Figure 1A and 1D), and were inversely associated with the tumor-node-metastasis (TNM) stage of GC patients (*P* < 0.001, Figure 1E). Additionally, deeper invasion (T3-4) tumor tissues showed reduced ALDOB levels than those with T1-2 (*P* < 0.05, Figure 1F). However, ALDOB expression did not exhibit any significant association with tumor size and location, or patients' gender (*P* > 0.05; Figure 2G-I).

The relationship between ALDOB expression and GC patients' clinicopathological manifestations was assessed (Table 1). Decreased ALDOB expression was associated with tumor invasion depth (χ^2^ = 5.918, *P* = 0.015), LNM (χ^2^ = 8.716, *P* = 0.003), and terminal stage of TNM (χ^2^ = 7.041, *P* = 0.008). However, its expression was associated with any other clinicopathologic features, like gender, age, and tumor size and location (*P* > 0.05, Table 1).

Additionally, Kaplan-Meier Plotter was utilized for investigating the ALDOB's role in the GC prognosis. As shown on the public website (https://kmplot.com), a low ALDOB mRNA expression was linked with shorter overall survival (OS), first progression (FP), and post-progression survival (PPS) (Figure 2A-C).

Altogether it was gathered that ALDOB was downregulated in GC and its decreased levels correlated with unfavorable clinicopathological manifestations and poor survival in patients.

### Stable knockdown and overexpression of ALDOB expression in GC cells

**Figure 3:**
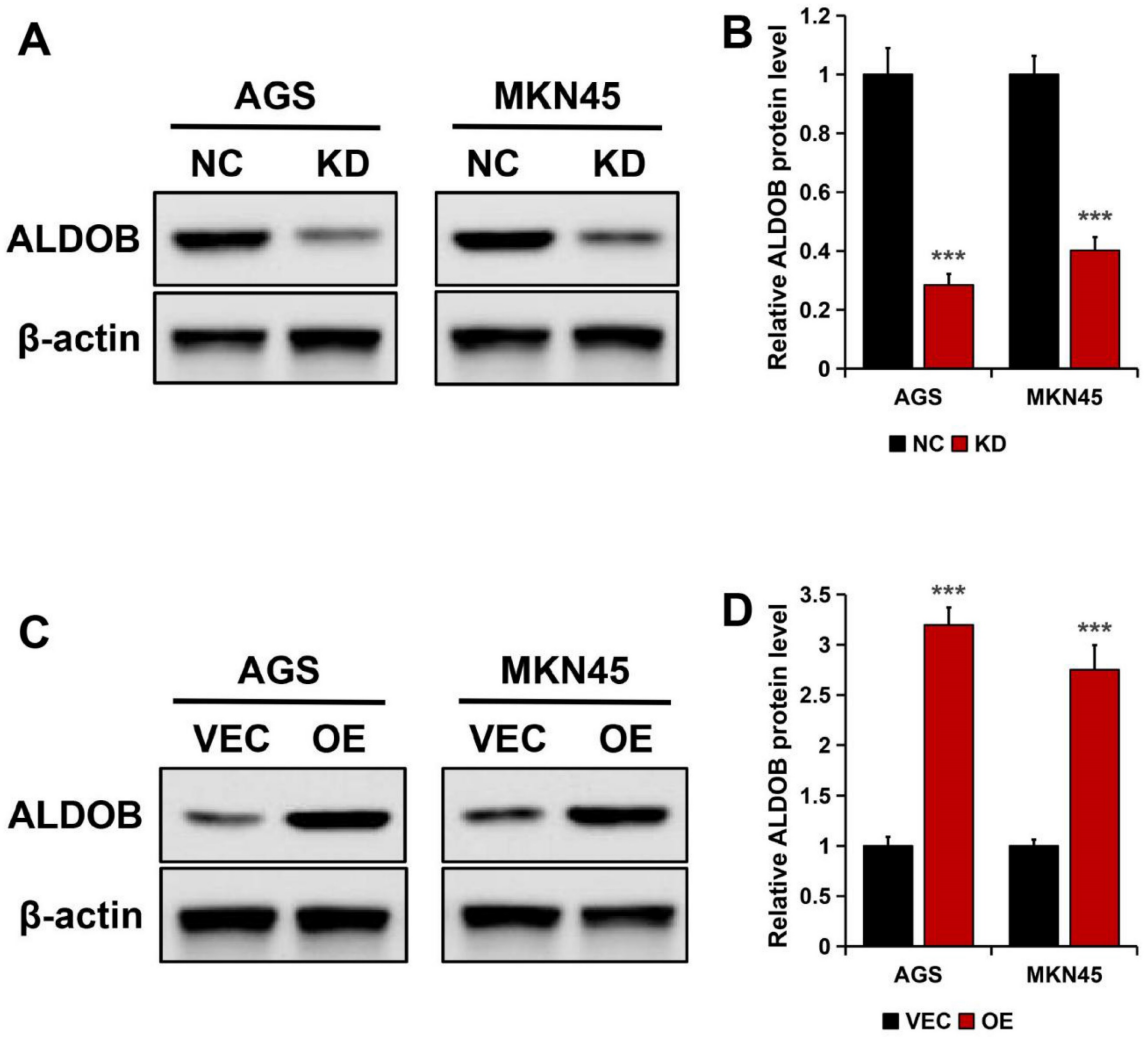
Knockdown and highly expressed ALDOB efficiency in GC cell lines. A. ALDOB protein expression in wild-type AGS and MKN45 and cells with stable ALDOB-KD. B. The quantified bands are presented as the mean ± SEM of three independent experiments. C. The ALDOB protein expression in wild-type AGS, MKN45 (VEC), and stable overly expressed ALDOB cells. D. The quantified bands are presented as the mean ± SEM of three independent experiments. β-actin was utilized as an endogenous control. ***P < 0.001.

In AGS and MKN45 cells, ALDOB was knockdown (KD) to generate transfectants, this was confirmed by Western blotting. The results revealed markedly reduced ALDOB protein expression in stable ALDOB-KD cells than the wild-type cells (NC) (*P* < 0.001; Figure 3A and B). For overexpressing ALDOB, human ALDOB (OE) encoding plasmids or empty vectors were transfected into GC cells and examined by Western blot assay, which revealed higher ALDOB protein expression level in plasmids encoding OE transfected cells than in empty vector cells (VEC) (*P* < 0.001; Figure 3C and D).

### ALDOB inhibits GC cell proliferation and migration

**Figure 4:**
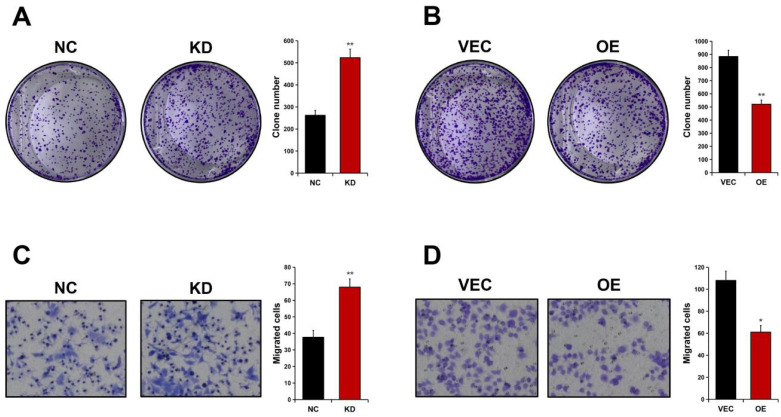
ALDOB suppresses the GC cell's ability to proliferate and migrate. A. Colony formation ability of ALDOB-KD AGS cells. B. Colony formation ability of ALDOB-overexpressed MKN45 cells. C. Migration ability of ALDOB-KD AGS cells and ALDOB-overexpressed MKN45 cells as detected by the Transwell assays. C. *P < 0.05, **P < 0.01.

Colony formation analyses were carried out to detect ALDOB's effect on GC cell proliferation. It was revealed that AGS cell's ability to form foci was significantly enhanced in absence of ALDOB (*P* < 0.01, Figure 4A), whereas elevated levels of ALDOB in MKN45 cells greatly impaired their colony-forming ability (*P* < 0.01, Figure 4B). To evaluate ALDOB's effect on GC cells' ability to migrate, Transwell migration analysis was conducted. The results indicated increased AGS cell migration ability after depleted ALDOB expression (*P* < 0.01, Figure 4C), and ALDOB overexpression in MKN45 cells showed inhibited migration capability (*P* < 0.05, Figure 4D). Altogether, ALDOB inhibits GC cells' ability to proliferate and migrate.

### ALDOB inhibits the migration and proliferation of GC cells via the AKT signaling pathway

**Figure 5:**
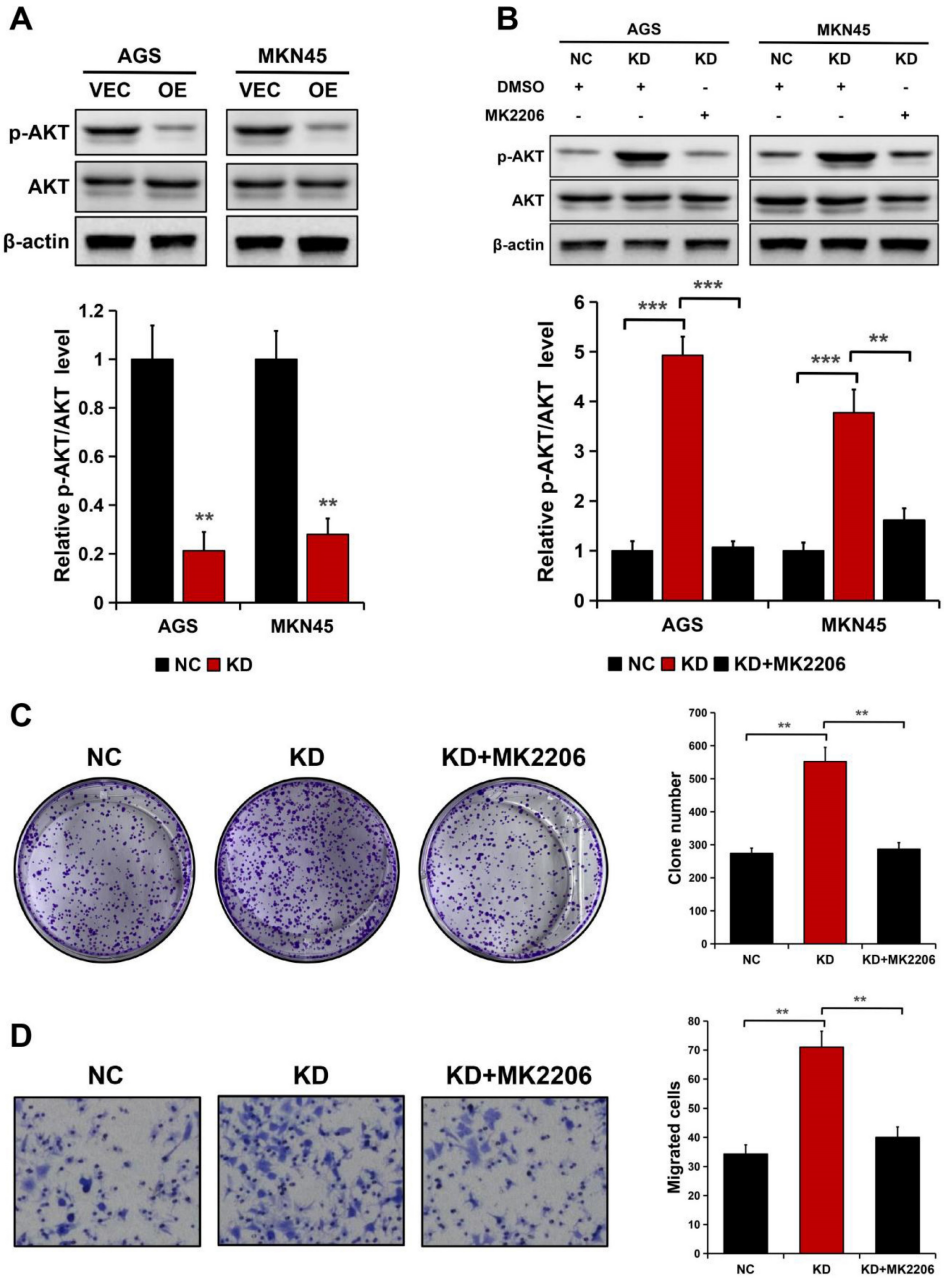
ALDOB suppresses the GC cell's ability to proliferate and migrate in an AKT-dependent manner. A. p-AKT and AKT expression in ALDOB-overexpressed cells assessed by Western blotting. B. p-AKT and AKT expression in ALDOB-KD cells with or without MK2206 treatment. C-D. Colony formation ability (C) and migration ability (D) of ALDOB-KD AGS cells with or without MK2206 treatment. β-actin was utilized as an endogenous control. **P < 0.01, ***P < 0.001.

Considering ALDOB negatively modulates AKT activation in HCC 19 and AKT is crucial for cancer cell's ability to proliferate and migrate 21, 22, it was hypothesized whether the AKT signaling pathway is implicated in ALDOB modulation of GC cell ability to proliferate and migrate. ALDOB overexpression notably decreased the phosphorylated AKT (p-AKT) expression in both cell lines (*P* < 0.01), but not the total AKT level (Figure 5A). Inversely, ALDOB depletion oppositely affected AKT phosphorylation (*P* < 0.01, Figure 5B). Subsequently, ALDOB-depleted AGS and MKN45 cells were treated with MK2206 (AKT suppressor). Western blots assessment indicated that MK2206 markedly inhibited AKT activation in these ALDOB-deficient cells (*P* < 0.05, Figure 5B). Additionally, colony formation assays (*P* < 0.001, Figure 5C) and migration assays (*P* < 0.05, Figure 5D) showed that cells' increased ability to proliferate and migrate induced by ALDOB-KD was partially impaired in cells under the influence of MK2206. Suggesting that ALDOB inhibits GC cells' ability to proliferate and migrate in an AKT-dependent manner.

## Discussion

It has been reported several times that enhanced aerobic glycolysis is one of cancer's hallmarks and cancerous cells develop certain characteristic alterations in glucose metabolism for supporting their unrestricted growth and metastasis 4, 5. Therefore, regulating specific metabolic mechanisms, for instance by targeting critical glycolytic enzymes, can be a promising strategy to treat cancer 4, 5. ALDOB is an essential part of glucose metabolism and reversibly catalyzes fructose-1,6-bisphosphate conversion into dihydroxyacetone phosphate and glyceraldehyde-3-phosphate 6, 7. In cancers, ALDOB's role is contentious as it can act as an anti-tumor or oncogenic enzyme. ALDOB was overexpressed in CRC tissues and was described as an oncogene in CRC 12-16. Whereas, it was reduced and acted as an anti-carcinogen in HCC 17-19. In GC tissues, ALDOB expression was decreased and its downregulation was positively linked with substandard prognosis in GC patients 20. However, the precise ALDOB function in GC cells and the related mechanisms are still undetermined and need further research.

This investigation provides evidence that ALDOB levels were decreased in GC tissues than in normal adjacent tissues, and was decreased more in LNM patients, terminal stage of TNM, and enhanced tumor size. Furthermore, analysis of clinical data also indicated that ALDOB levels markedly correlated with the tumor invasion depth (*P* = 0.015), LNM (*P* = 0.003), and TNM stage (*P* = 0.008). The loss- and gain-of-function assessment demonstrated that ALDOB inhibited GC cells' capability to proliferate and migrate, suggesting its' anti-tumor activity in GC.

AKT, a cell survival regulator, is observed to be hyper-activated and highly expressed in various human cancers including GC 21-23. Since ALDOB negatively modulates AKT activation 19, it was hypothesized that ALDOB's inhibitory effect on GC cell growth and metastasis can be dependent on AKT. It was discovered that ALDOB-KD increased AKT phosphorylation while its overexpression had the opposite impact. Additionally, the enhanced growth and metastasis induced by ALDOB-KD were impaired partially in cells treated with MK2206, suggesting the involvement of AKT in the ALDOB-mediated GC cell inhibition of growth and migration.

Recently, a study uncovered the physiological significance of ALDOB-induced AKT inactivation via ALDOB/AKT/PP2A protein complex formation, relying upon the enzymatic activity of ALDOB 19. In normal cells, the interaction between ALDOB and AKT is in an AKT kinase activity-dependent manner, which stimulates the interaction of phosphatase PP2A with p-AKT and causes dephosphorylation of p-AKT. ALDOB modulates intracellular homeostasis of AKT phosphorylation by regulating interactions between substrates and phosphatase. In cancer cells deprived of ALDOB, there is inhibiting modulation of AKT activity, which causes essential stimulation of oncogenic AKT signaling, thereby, facilitating the progression of the cell cycle and increasing glucose exhaustion and glycolysis flux for cancer cell growth and metastasis. The investigation demonstrates that when hyperactive AKT is targeted by direct inhibition of AKT activity or by reactivating PP2A phosphatase, it may act as a potential treatment strategy for ALDOB-deficient human cancers.

In total, the data from this investigation indicated the clinical importance of ALDOB in GC and also established its suppressing function in GC cells' ability to proliferate and migrate. Furthermore, it was also discovered that ALDOB inhibited GC cell growth and metastasis partially by participating in AKT signaling pathway inhibition. Therefore, ALDOB can act as a potent and beneficial target for future GC treatment.

## Figures and Tables

**Table 1:** Relationships between ALDOB expression and clinic-pathological characteristics of GC patients

Characteristics	Number of cases	ALDOB expression		χ^2^	P value
		None or low	High		
**Total**	79	40 (50.6%)	39 (49.4%)		
**Age**					
<65	42	25 (59.5%)	17 (40.5%)	2.836	0.092
>=65	37	15 (40.5%)	22 (59.5%)		
**Gender**					
Male	51	25 (49.0%)	26 (51.0%)	0.150	0.699
Female	28	15 (53.6%)	13 (46.4%)		
**Tumor size**					
<5cm	40	17 (42.5%)	23 (57.5%)	2.144	0.143
>=5cm	39	23 (59.0%)	16 (41.0%)		
**Tumor location**					
U	43	21 (48.8%)	22 (51.2%)	0.122	0.727
L	36	19 (52.8%)	17 (47.2%)		
**Depth of invasion**					
T1-2	19	5 (26.3%)	14 (73.7%)	5.918	0.015*
T3-4	60	35 (58.3%)	25 (41.7%)		
**Lymph node metastasis**					
No	26	7 (26.9%)	19 (73.1%)	8.716	0.003**
Yes	53	33 (62.3%)	20 (37.7%)		
**TNM Stage**					
I/II	29	9 (31.0%)	20 (69.0%)	7.041	0.008**
III/IV	50	31 (62.0%)	19 (38.0%)		

*P < 0.05, **P < 0.01
